# Bone Metastases from Gastrointestinal Stromal Tumor: A Case Report

**DOI:** 10.1155/2012/509845

**Published:** 2012-12-03

**Authors:** Jihen Feki, Racem Bouzguenda, Lobna Ayedi, Moez Bradi, Tahia Boudawara, Jamel Daoud, Mounir Frikha

**Affiliations:** ^1^Oncology Department, Habib Bourguiba University Hospital, El Ain Road, 3029 Sfax, Tunisia; ^2^Pathology Department, Habib Bourguiba University Hospital, El Ain Road, 3029 Sfax, Tunisia; ^3^Radiology Department, Habib Bourguiba University Hospital, El Ain Road, 3029 Sfax, Tunisia; ^4^Radiotherapy Department, Habib Bourguiba University Hospital, El Ain Road, 3029 Sfax, Tunisia

## Abstract

Gastrointestinal stromal tumors (GISTs) are the most common mesenchymal neoplasms of the gastrointestinal tract. Their most common metastatic sites are the liver and the peritoneum, but GISTs rarely metastasize to the bones. We report a case of a 58-year-old man with sternoclavicular joint metastasis from a GIST manifesting 28 months after surgical resection of the small intestine tumor. We will discuss through this paper and a literature review the clinical characteristics, imaging features, and management of this unusual metastatic location of GIST.

## 1. Introduction

Gastrointestinal stromal tumors (GISTs) are the most common mesenchymatous tumors of the gastrointestinal tract [1]. They most commonly occur at the age of over 50 years [2]. In our oncology center of Sfax, we diagnosed thirty cases of GISTs between 2000 and 2010. The most frequent anatomic location of GIST is the stomach (60%) and the small intestine (30%). Five to ten percent of GISTs arise from the colon and rectum, less than 1% are located in the esophagus, and it can originate beyond the gastrointestinal tract in the omentum, mesentery or retroperitoneum [3].

Their most common metastatic sites are the liver and the peritoneum, but GISTs rarely metastasize to the bones. We will discuss through this paper and a literature review the clinical characteristic, imaging features, and management of this unusual metastatic location of GIST.

## 2. Case Report

**Figure 1:**
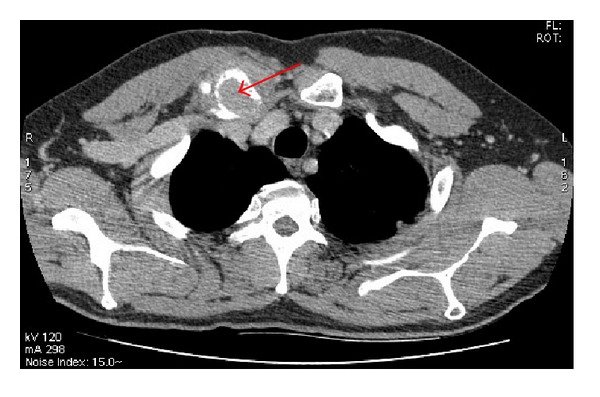
Bone metastasis in the sternoclavicular joint invading adjacent soft tissues.

**Figure 2:**
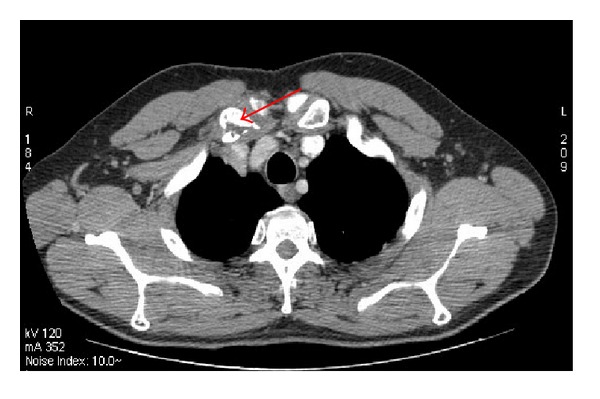
Partial response after radiotherapy and 10 months of imatinib with partial reossification in the sternoclavicular joint and decreasing of the soft tissue extension.

**Figure 3:**
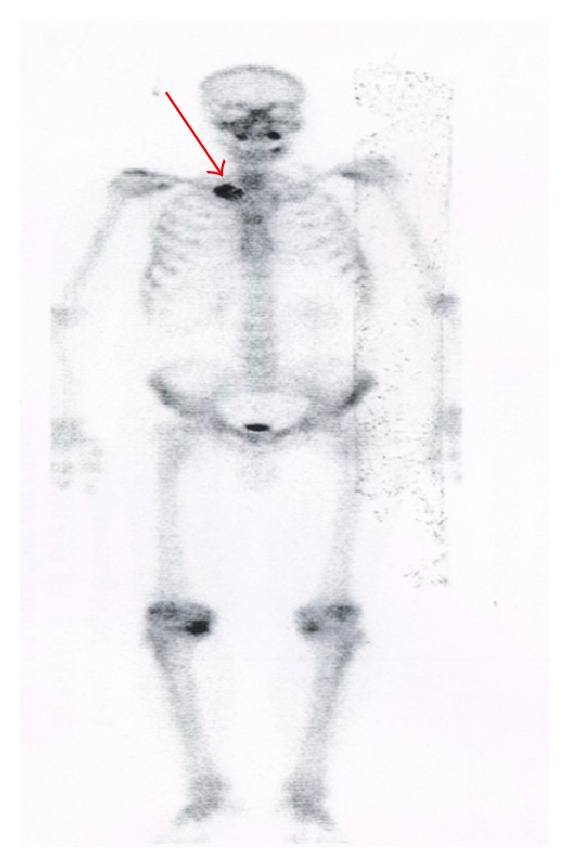
Bone scintigraphy image showing increasing intensity of tracer uptake localized in the sternoclavicular joint.

**Figure 4:**
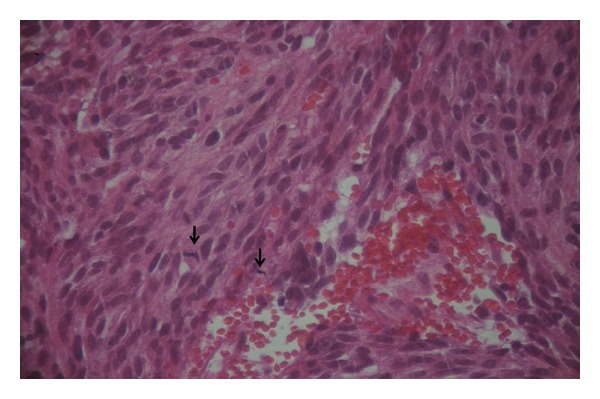
Bone marrow biopsy: Spindle cells with high cellularity. Some of the tumor cells show mild pleomorphism. Numerous mitotic figures are present (↘) (hematoxylin and eosin: ×400).

**Figure 5:**
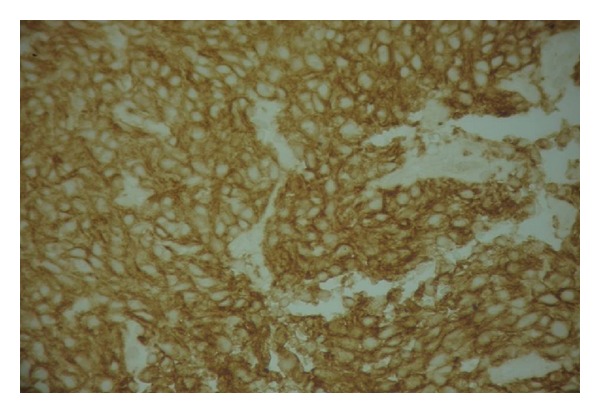
Positive immunostaining with c-Kit (CD117). Note diffuse membranous pattern (HE ×400).

A 58-year-old man had undergone surgical removal of a small intestine tumor in January 2008. Histological examination showed a gastrointestinal stromal tumor with a 16 cm primary tumor in the jejunum. Immunohistochemistry revealed spindle cells positive for CD117 and CD34. Mitotic activity was low. No adjuvant treatment was available at that time. The patient was clinically stable and followed by serial imaging until May 2010, when he presented a painful sternoclavicular joint tumefaction. The thoracoabdominal CT scan revealed an osteolytic sternoclavicular joint mass (Figure 1) and 3 liver metastases. Bone scintigraphy showed an increasing intensity of tracer uptake localized in the sternoclavicular joint (Figure 3). The patient underwent a biopsy of the sternoclavicular joint mass. Immunohistochemical staining demonstrated the tumor cells to be positive for c-kit (CD117) (Figure 5) protein and CD34 protein. The histological features and staining pattern of the tumor cells were consistent with a GIST metastasis (Figures 4 and 5). Our patient received radiation therapy at the sternoclavicular joint with a total dose of 30 grays. Afterward he began treatment with oral imatinib mesylate at a dose of 400 mg/day. The clinical response was good and the patient's pain resolved. Control contrast-enhanced CT scans showed a partial response in bone lesion (Figure 2) as well as in liver metastases.

After 19 months, the patient complained of weakness of lower limbs. Our patient refused vertebromedullary magnetic resonance imaging. A thoracoabdominal CT scan showed 2 metastatic osteolytic lesions of T1 and T10 vertebral body with spinal cord invasion. Urgent radiotherapy was directed at the T1 and T10 vertebra but stopped at the dose of 15 grays because of an affected general state. Our patient kept definitive paraplegia. 

## 3. Discussion

GISTs are defined as pleomorphic mesenchymal tumors of the gastrointestinal tract composed of spindle cells, epithelioid cells, or a combination of both that express the KIT protein (CD 117) and in most cases CD34 on immunohistochemistry [4, 5]. 

GISTs have an uncertain clinical behavior ranging from benign to frankly malignant, making the outcome totally unpredictable. Over the years, many factors have been examined [1]. Current guidelines categorize GISTs as low, intermediate, and high risk based on size, mitotic index and anatomic location [3]. In our case, because the tumor size was >10 cm, with rare mitoses (less than 5 per 50 HPF) and the jejunal location, the tumor had a high risk of metastases or tumor-related death reaching 52% [3]. Metastasis is characteristically the malignant behavior of the GIST. Overall, approximately 10–30% of GISTs exhibit malignant behavior [1]. There are only a few reported cases in the literature of patients with GIST metastases to the bone [6, 7]. Bertulli et al. reported that thirteen out of 278 patients (5%) with GIST had bone metastases which was the only metastasization site in 4 cases or associated with other metastases in 9 cases [8]. In the study of Jati et al., of 190 GIST patients, six (3.2%) had bone metastases [9]. 

Bone metastases can be diagnosed rarely at disease presentation and more frequently at disease relapse [10]. The most frequent sites of bone metastases reported in many case series were spine and pelvis [8, 10].

Clinically, bone metastases are mostly symptomatic and revealed by bone fractures or bone pain as our patient or spin cord compression syndrome but they can be asymptomatic and are an incidental finding of the occasion of the practice of CT or PET-SCAN [11]. 

In our case, all bone lesions were osteolytic with invasion to the adjacent soft tissues. Di Scioscio described the radiologic aspect of bone metastases in a series of 3 cases. The lesions were mostly lytic, with a complete rearrangement of bone structure, cortex erosion, and, in one case, a solid mass invading adjacent soft tissues [10]. 

Few data can be found in the literature on the treatment of bone metastases in GISTs. The use of imatinib mesylate in recurrent or metastatic, resectable or not GIST in prospective trial has shown response in 50% patients, and in approximately 75–85% patients have at least stable disease. Imatinib mesylate has proven also efficacy in bone metastases of GIST [4]. Nineteen months of response were recorded in this paper. Bertulli et al. reported that median survival of 13 patients with GIST metastases to the bone was 17 months (3–40 m) [8]. 

GISTs are not considered as a radiosensitive tumor [12], but radiotherapy can be used for palliative purposes. Chou et al. described a case of 82-year-old woman with T3 vertebral body metastasis of a vulval GIST with spinal cord invasion causing bilateral lower limb weakness. After radiotherapy, the patient's lower limbs regained near-full power [13]. In our case, radiotherapy allowed an excellent analgesic effect in the sternoclavicular joint lesion. 

The effect of biphosphonate on GIST's bone lesions is unknown, even though it may be recommended [10]. 

## 4. Conclusion

Bone metastases from GISTs are rare, and there are only a few reported cases in the literature, but they may become more prevalent due to increased patient life expectancy as well as the improvement in imaging techniques and they should always be sought. Imatinib is also an effective treatment in case of bone metastatic GISTs as was demonstrated in this case paper. Radiation therapy can be discussed in palliative indication.
